# Power to the voice hearer — The German version of the voice power differential scale

**DOI:** 10.1371/journal.pone.0230778

**Published:** 2020-03-26

**Authors:** A. Gmeiner, A. Gaglia, S. Habicher, T. Rumpold, S. Süßenbacher, B. Schrank, M. Amering

**Affiliations:** 1 Division of Social Psychiatry, Department of Psychiatry and Psychotherapy, Medical University of Vienna, Vienna, Austria; 2 Division of Psychology, Bangor University Wales, Bangor, United Kingdom; 3 Department of Adult Psychiatry, Karl Landsteiner University for Health Sciences, University Clinic Tulln, Vienna, Austria; Universite de Bretagne Occidentale, FRANCE

## Abstract

Voice power is an important concept in daily life of voice hearers and in the support and therapy for voice hearers who seek help. Therefore, the ability to examine voice power differentials between a voice and a voice hearer is essential. The present study aimed to collect data on voice power differentials and to further validate the Voice Power Differential Scale (VPD). 105 participants aged ≥ 18 with an ICD10 F2-diagnosis that included hearing voices were included in this study. Internal consistency was good (alpha = 0.792), as well as test-retest-reliability (r = 0.855) and correlations with other constructs were generally as expected. The VPD questionnaire results correlated negatively with the Beliefs About Voices Questionnaire-Revised’s (BAVQ-R) items of Benevolence and Engagement-emotion. It correlated positively with Omnipotence and Resistance-emotion, as well as with Negative Content on the Psychotic Symptoms Rating-Scale (PSYRATS). Unexpectedly, no correlations were found with overall severity and command hallucinations. The Voice Power Differential Scale is an important tool for assessing and formulating a voice hearer’s experience when they seek treatment or support for their verbal auditory hallucinations. The results of this study enrich the on-going discussion about the importance of voice power for voice hearers.

## Introduction

The experience of hearing voices is varied. Hearing voices can occur in the general population [1–3], with many people reporting that voice hearing has been a meaningful human experience [4, 5]. Additionally, hearing voices is common among people with a diagnosis of a schizophrenia spectrum disorder with approximately 70% of people with this diagnosis experiencing this phenomenon[6]. In the context of schizophrenia, voices are labeled as verbal auditory hallucinations (VAH) and are understood to be amongst the most treatment resistant symptoms [7], while they are often stated within treatment resistant schizophrenia [8, 9]. A promising area of development over the last years for voice hearers who are distressed by their experiences is in the field of cognitive behavioural therapy (CBT), e.g. Paulik et al. [10]. Through the application of the cognitive model to verbal auditory hallucinations [11] power differentials have emerged as important concepts in the voice–voice hearer relationship [12] and the perceived power of voices and subjective control over voice hearers’ experiences is one of the key processes targeted by psychological interventions [13].

Higher perceived voice power or voice omnipotence has been shown to be associated with the amount and intensity of voice related distress [14–16] as well as with depression, suicidal thoughts and higher social distress [14, 17]. Voice power was found to be one of the best predictors of harmful compliance among people diagnosed with schizophrenia, schizo-affective, mood-disorders [18] and a forensic sample with mixed diagnoses [19]. Voice hearers who experience their voice as dominant, malevolent and frowning suffer from higher burden and have been shown to be at greater risk of engaging in aggressive and self-harming behaviours [20–22].

While power relationships play a central role in different instruments that assess VAHs, such as the item Controllability of the Psychotic Symptoms Rating Scale [23, 24] and the item Omnipotence in the Beliefs About Voices Questionnaire [25], the Voice Power Differential Scale (VPD) [26] focuses exclusively on the power relationship. The VPD was originally validated with good psychometric properties in a sample of 59 people diagnosed with schizophrenic psychoses. It has since been used in studies on CBT [14, 27–29] and in an evaluation of AVATAR-therapy [30] in patients with a diagnosis of schizophrenia spectrum disorders as well as mixed-diagnosis samples, and in a sample diagnosed with Borderline Personality Disorder [31].

The study described here aimed to further validate the VPD through a German language version tested on a sizeable sample of people with schizophrenia spectrum disorders.

## Materials and methods

### Procedure

Our working group translated the original English version of the VPD to German, which was then translated back into English by a bilingual native-speaker. The German version and its back translation were then reviewed and approved by the author of the original version [32].

Study inclusion criteria were: males or females ≥ 18 years old, who heard voices in the 4 weeks prior to recruitment, who also met criteria for an ICD-10 F2-diagnosis [33] and were willing to consent to participation. Exclusion criteria were: an insufficient knowledge of the German language and people who were in hospital as involuntary patients.

The Ethics Committee of the Medical University Vienna provided approval for the study (EC-number: 1342/2013). Study participants were recruited from the Department of Psychiatry and Psychotherapy at the Medical University of Vienna in-patient and day-clinics, the University Clinic for Psychiatry and Psychotherapy Tulln’s in-patient department, and the Social Psychiatric Center of the Caritas Vienna’s day-clinic. Potential study participants were informed about the study by their clinical teams who subsequently referred interested people to the research-team. Potential participants were then provided with the details and requirements of the study and those who wished to participate in the study provided written informed consent in accordance with the guidelines of the Ethics Committee of the Medical University of Vienna. A member of the research team (all with a medical background) then met with a potential study participant to take informed consent and to distribute the questionnaires which were self-report. A researcher was present while participants completed the questionnaires to answer any questions. Depending on the preference of the participant, the researcher met with the participant at the clinic or at the participant’s home.

### Instruments

#### Socio-demographic and clinical variables

Demographic and clinical variables including age, sex, family status, social network, education, job status, living arrangements, time elapsed since first diagnosis, age at first in-patient admission, number of in-patient stays and duration of voice hearing experience were gathered using a self-report questionnaire.

#### Voice power differential—Scale (VPD)

The VPD [26], comprised of a 5-point-Likert-scale measures the power differential between voice and voice hearer on 7 dimensions: power, strength, confidence, respect, ability to inflict harm, superiority and knowledge. The greater the score, the greater the perceived power differential, e.g. “I am much stronger than my voice” [1]–“My voice is much stronger than me” [5]. Participants who heard more than one voice were asked to complete the questionnaire with respect to their experience of the voice that they perceived to be most dominant. The scale showed good internal reliability (Cronbach's α = 0.85) with high test-re-test reliability (r = 0.82) in the original study that established the measure’s validity [26].

#### The Beliefs About Voices Questionnaire–Revised (BAVQ-R)

The BAVQ-R [25], often used in CBT, is an instrument that assesses beliefs about voices as well as behavioural and emotional responses to them on 5 subscales: malevolence, benevolence, omnipotence, resistance and engagement. The BAVQ-R consists of 35 items that are self-rated on a 4-point-Likert-scale (0 = disagree, 1 = don’t know, 2 = rather agree, 3 = agree), again this scale was completed by the participants for their dominant voice. The German-language version [17] showed high internal consistency for the subscales malevolence (a = 0.83), benevolence (a = 0.91), resistance (a = 0.85), engagement (a = 0.87) but a low internal consistency in the subscale omnipotence (a = 0.62). Test-Retest-Reliability was satisfactory.

#### Psychotic symptoms rating scale–auditory hallucinations (PSYRATS-AH)

PSYRATS-AH [23, 24, 34], also often used in CBT, is a reliable and valid semi-structured interview that inquires about the participants’ experience of their voices overall. It consists of eleven items covering the following dimensions: frequency, duration, location, loudness, beliefs of origin of voices, amount of negative content, amount and intensity of distress, life-disruption, and controllability of the voices. Data are assessed on a 5-point ordinal scale (0–4).

#### Additional items

These participants of this group of people under medical treatment were also asked if they ever self-harmed, harmed anyone else, or attempted suicide in the past. Additionally, they were asked if the voices ever commanded them to harm themselves or someone else.

#### Clinical global impression–schizophrenia scale (CGI-SCH)

The CGI [35] was developed by Busner & Targum and adapted for schizophrenia (CGI-SCH) by Haro et al [36]. Used in routine clinical practice as well as research, the brief, valid and reliable instrument, assesses the symptom severity of the following five items on a seven-point-Likert-scale: positive symptoms (CGI-pos), negative symptoms (CGI-neg), depressive symptoms (CGI-dep), cognitive symptoms (CGI-cog) and overall severity (CGI-total).

### Statistical analysis

All statistical analyses were calculated with “Statistical Package for Social Sciences”(SPSS Version 24, SPSS GmbH, IBM AG, USA). Descriptive statistics are presented in absolute numbers and sample percentages. All analyses were performed using two-tailed tests with alpha = 0.05. In order to analyse reliability, Cronbach’s alpha was calculated with values higher than 0.7 were considered acceptable. The test-retest-reliability was calculated via Pearson correlations, again values above 0.7 were considered acceptable. Construct validity was calculated via Pearson-correlations with PSYRATS-AH, BAVQ-R and CGI-SCH. A factor analysis of the VPD was not conducted in this study due to the small number of items.

## Results and discussion

### Recruitment

A total of 130 eligible participants were referred to the research team of which 105 provided informed consent and completed the questionnaires. A sample size of 105 is in compliance with the conventional rule for validation studies [37]. In order to assess the test-retest-reliability of the VPD, 19 participants were recruited to repeat the VPD after 7–15 days (mean: 9d ± 2.2).

### Study sample

Socio-demographic and clinical data are shown in Table 1. 56.2% of the participants were male, with a median age of 33.0 years (range: 19.0–86.0) and a median duration of voice hearing experience of 10.5 years (range: 0.1–45.0). 79% of the participants were single, 42.9% were out of the workforce as either retired or in receipt of permanent disability benefits, and only 3.8% were employed. 32.4% had a sufficient social network (self-defined by each participant), 49.5% lived alone in their own household. These socio-demographic data are typical for a sample with diagnoses of schizophrenia spectrum disorders [38].

**Table 1:** Sociodemographic data.

Median age in years (range)	33.0	(19.0–84.0)
Median age in years at first diagnosis (range)	21.0	(4.0–48.0)
Median duration of voice hearing experience in years (range)	10.5	(0.1–45.0)
**Gender**	**N**	**%**
Female	46	43.8%
Male	59	56.2%
**Family status**		
Single	83	79.0%
Married	10	9.5%
Divorced or separated	12	11.4%
**Social network (self-defined by each participant)**		
None or little	24	22.9%
Short-term acquaintances	18	17.1%
Few friends	29	27.6%
Sufficient	34	32.4%
**Living arrangements**		
With parents	19	18.1%
Own household (with partner etc.)	19	18.1%
Own household alone	52	49.5%
Shared accommodation	7	6.7%
Supervised living	7	6.7%
Missing	1	1.0%
**Working status in current or last job**		
Apprentice	7	6.7%
Unskilled worker	18	17.1%
Skilled worker	13	12.4%
Employee	33	31.4%
Employee, leading position	3	2.9%
Self-employed	1	1.0%
Freelance	3	2.9%
Other	25	23.8%
Missing	2	1.9%
**Current working situation**		
Employed / sick leave	4	3.8%
Unemployed / sick leave	14	13.3%
Retired / disability pension	45	42.9%
Homemaker	1	1.0%
Student	5	4.8%
Minimum income	13	12.4%
Unemployment benefit	11	10.5%
Other	11	10.5%
Missing	1	1.0%
**Highest education (no mandatory completion)**		
Special needs school	3	2.9%
Compulsory school	12	11.4%
Vocational school	35	33.3%
Middle school	32	30.5%
University	22	21.0%
Missing	1	1.0%

### Reliability

#### Internal consistency

Internal consistency was good at 𝜶 = 0.792.

#### Test-retest-reliability

To examine test-retest-reliability of the VPD, 19 participants completed the VPD-scale again after 7–15 days (SD ± 2.24). Good test-retest-reliability with a Pearson’s correlation coefficient of 0.855 (p ≤ 0.001) was shown.

### Construct validity

With regards to construct validity, the following expected correlations were shown: there were negative correlations between VPD overall and the BAVQ-R subscales Benevolence (r = -0.268, p = 0.009) and Engagement-Emotion (r = -0.294, p = 0.004), and positive correlations between VPD overall and the BAVQ-R subscales Omnipotence (r = 0.485, p ≤ 0.001), Resistance-Emotion (r = 0.295, p = 0.004). The VPD item “pure power” correlated negatively with the subscale Engagement–Emotion (r = -0.093, p = 0.019) and positively with the subscales Malevolence (r = 0.237, p = 0.018), Omnipotence (r = 0.504, p ≤ 0.001) and Resistance-Emotion (r = 0.028, p = 0.002). The VPD item strength correlated negatively with Benevolence (r = -0.235, p = 0.018), Engagement-Emotion (r = -0.233, p = 0.019) and positively with Omnipotence (r = 0.439, p ≤ 0.001) and Resistance-Emotion (r = 0.277, p = 0.005). Confidence correlated positively with Omnipotence (r = 0.360, p ≤ 0.001) and Resistance-Emotion (r = 0.203, p = 0.044). Ability to inflict harm correlated negatively with Benevolence (r = -0.318, p = 0.002), Engagement-Emotion (r = -0.397, p ≤ 0.001) and Engagement-Behaviour (r = -0.205, p = 0.046) and positively with Omnipotence (r = 0.289, p = 0.005). Superiority correlated negatively with Benevolence (r = -0.216, p = 0.032) and Engagement-Emotion (r = -0.245, p = 0.014), and positively with Omnipotence (r = 0.303, p = 0.003). Knowledge correlated negatively with Resistance-Behaviour (r = -0.212, p = 0.036) and positively with Omnipotence (r = 0.228, p = 0.025). Details about correlations between BAVQ-R and VPD are shown in Table 2. Finally, calculations based on the 4 BAVQ-R factors recently identified by Strauss et al. [39] showed the following results: The factor Persecutory Beliefs correlated positively with Power (r = 0.313, p = 0.002) and Strength (r = 0.228, p = 0.024). The factor Benevolence correlated negatively with Strength (r = -0.249 p = 0.012), Ability to inflict harm (r = -0.339, p = 0.001) and Superiority (r = -0.226, p = 0.025). The factor Resistance did not show any significant correlations. The factor Engagement correlated negatively with Strength (r = -0.244, p = 0.014), Ability to inflict harm (r = -0.342, p = 0.001) and Superiority (r = -0.277, p = 0.006).

**Table 2:** Correlations between VPD and BAVQ-R.

	VPD	Power	Strength	Confidence	Respect	Ability to inflict harm	Superiority	Knowledge
Malevolence	r = 0.114	r = 0.237	r = 0.143	r = 0.033	r = 0.173	r = 0.088	r = 0.043	r = -0.091
	p = 0.275	**p = 0.018**	p = 0.156	p = 0.747	p = 0.092	p = 0.397	p = 0.671	p = 0.375
Benevolence	r = -0.268	r = -0.110	r = -0.235	r = -0.106	r = -0.091	r = -0.318	r = -0.216	r = -0.091
	**p = 0.009**	p = 0.274	**p = 0.018**	p = 0.292	p = 0.377	**p = 0.002**	**p = 0.032**	p = 0.374
Omnipotence	r = 0.485	r = 0.504	r = 0.439	r = 0.360	r = 0.189	r = 0.289	r = 0.303	r = 0.228
	**p ≤ 0.001**	**p ≤ 0.001**	**p ≤ 0.001**	**p ≤ 0.001**	p = 0.066	**p = 0.005**	**p = 0.003**	**p = 0.025**
Resistance–*Emotion*	r = 0.295	r = 0.028	r = 0.277	r = 0.203	r = 0.200	r = 0.200	r = 0.165	r = -0.008
	**p = 0.004**	**p = 0.002**	**p = 0.005**	**p = 0.044**	p = 0.051	p = 0.052	p = 0.105	p = 0.939
Resistance—*Behaviour*	r = -0.092	r = 0.028	r = -0.049	r = -0.077	r = -0.031	r = 0.047	r = -0.172	r = -0.212
	p = 0.373	p = 0.781	p = 0.627	p = 0.947	p = 0.761	p = 0.647	p = 0.088	**p = 0.036**
Engagement–*Emotion*	r = -0.294	r = -0.093	r = -0.233	r = -0.119	r = -0.126	r = -0.397	r = -0.245	r = -0.072
	**p = 0.004**	**p = 0.019**	**p = 0.019**	p = 0.237	p = 0.218	**p ≤ 0.001**	**p = 0.014**	p = 0.481
Engagement—*Behaviour*	r = -0.151	r = -0.024	r = -0.088	r = -0.052	r = -0.018	r = -0.205	r = -0.183	r = -0.088
	p = 0.145	p = 0.814	p = 0.383	p = 0.608	p = 0.859	**p = 0.046**	p = 0.072	p = 0.392

Respect correlated negatively with the CGI item Negative Symptoms and positively with the item Depressive Symptoms (r = 0.214, p = 0.037). No correlations were shown with regards to overall severity. Details about correlations between CGI and VPD are shown in Table 3.

**Table 3:** Correlations between VPD and CGI-SCH.

	VPD	Pure power	Strength	Confidence	Respect	Ability to inflict harm	Superio-rity	Knowledge
CGI	r = 0.016	r = 0.042	r = 0.033	r = 0.116	r = 0.138	r = -0.153	r = -0.001	r = -0.307
	p = 0.897	p = 0.685	p = 0.744	p = 0.259	p = 0.182	p = 0.141	p = 0.991	p = 0.721
CGI–pos.	r = 0.016	r = 0.063	r = 0.132	r = 0.133	r = 0.023	r = -0.172	r = -0.05	r = 0.016
	p = 0.880	p = 0.539	p = 0.195	p = 0.194	p = 0.822	p = 0.097	p = 0.593	p = 0.876
CGI–neg.	r = -0.030	r = 0.016	r = 0.008	r = 0.007	r = 0.151	r = -0.219	r = -0.033	r = -0.036
	p = 0.776	p = 0.878	p = 0.937	p = 0.945	p = 0.144	**p = 0.034**	p = 0.748	p = 0.730
CGI–dep.	r = -0.045	r = 0.018	r = 0.023	r = -0.057	r = 0.214	r = -0.196	r = -0.140	r = -0.112
	p = 0.670	p = 0.862	p = 0.826	p = 0.597	**p = 0.037**	p = 0.059	p = 0.171	p = 0.276
CGI–cog.	r = - 0.009	r = 0.061	r = -0.012	r = 0.140	r = 0.051	r = -0.194	r = -0.006	r = 0.006
	p = 0.935	p = 0.554	p = 0.904	p = 0.171	p = 0.625	p = 0.061	p = 0.954	p = 0.956

43.8% perceived voices that commanded self-harm, 24.8% perceived voices that commanded to harm others. 30.5% had ever harmed themselves, 23.8% had attempted suicide. These demographics are in line with those reported for clinical voice hearers in the meta-analysis by Baumeister et al. [2]. Self-harming or harming behaviour and VPD were not shown to be correlated.

The PSYRATS-item Duration of voices correlated positively with power (r = 0.238, p = 0.017) and strength (r = 0.304, p = 0.002). Loudness of the voice correlated negatively with the VPD item Ability to inflict harm (r = -0.207, p = 0.043). Negative content correlated positively with VPD overall (r = 0.250, p = 0.014), Strength (r = 0.217, p = 0.029) and Superiority (r = 0.266, p = 0.007). Calculations based on the 4 factors identified by Woodward et al. [40] showed the following results: The factor Distress correlated positively with Power (r = 0.220, p = 0.029), Strength (r = 0.258, p = 0.010) and Superiority (r = 0.288, p = 0.004). The factor Frequency correlated positively with Power (r = 0.212, p = 0.035) and Strength (r = 0.271, p = 0.006). The Attribution factor did not show any significant correlations. The factor Loudness correlated negatively with Ability to inflict harm (r = -0.207, p = 0.043).

### Descriptive statistics of the VPD

The VPD yields a score of between 7–35 with higher scores indicating greater power for the voice relative to the voice hearer [41]. The mean of the VPD score for these study participants was 23.3, which was relatively high. The means of each dimension, with the exception of the dimension “Strength” which was 3.0, were above 3, indicating that voice power was rather high. About 90% of the participants completed the VPD-scale fully and within 2 minutes. Details are shown in Table 4.

**Table 4:** Descriptive statistics of the VPD.

Items	N	Mean	Standard Deviation ±	Missing in %
VPD-total	96	23.3	5.97	8.6
Power	101	3.22	1.30	3.8
Strength	102	3.00	1.30	2.9
Confidence	101	3.18	1.36	3.8
Respect	98	3.34	1.11	6.7
Ability to inflict harm	97	3.72	1.21	7.6
Superiority	100	3.36	1.23	4.8
Knowledge	99	3.30	1.37	5.7

105 persons with a diagnosis of a schizophrenia spectrum disorders and voice hearing experience participated in this largest study on the validity of the Voice Power Differential Scale [26] to date. The VPD was fast and easy to complete and had a 90% total completion rate.

The descriptive statistics of the VPD-scale (overall-score) showed a mean of 23.3. In the domains “power”, “confidence”, “respect”, “ability to inflict harm”, “superiority”, and “knowledge”, the voice was experienced to be more powerful than the voice hearer whereas the item “strength” showed an equal power balance. This is consistent with the original study in which 59 people diagnosed with schizophrenic psychoses were included [26] and two further studies [15, 27], in which the means of the total-scale were even higher. These results suggest voice hearers tend to experience their voices as more potent than themselves, thus voice hearers are likely to experience themselves as overpowered, bullied, or scared. Regardless of whether the main focus is on the concepts of voice content [42], contribution to goal interference and facilitation [43], or specialised and non-specialised therapies for alleviating the effects of voices [44], voice power is an essential construct.

The Internal Consistency (Cronbach’s Alpha: 0.792) was good, but lower than in the original study [26] and two further studies assessing reliability [27, 45]. Test-Retest-Reliability was good and comparable to the original study [26]. Therefore, it can be assumed that the power differential between voice hearer and voice is stable over a couple of weeks and that the results do not depend on daily conditions.

In order to determine the construct validity, correlations between the power of the voice, beliefs about voices (BAVQ-R), voice topography (PSYRATS-AH), and severity of positive, negative, depressive, and cognitive symptoms (CGI-SCH) were calculated. The original validation study [26] found a correlation between voice power and depressive symptoms, measured on the Beck Depression Inventory. The current study demonstrated a positive correlation between depressive symptoms and voice power subscale of respect. In this study, the loudness and duration of voice were related to voice power. In the original study, loudness was found to be related as well. However, loudness is not a clearly defined construct and it would benefit from additional research to further investigate its importance. In addition, this study found negative content and voice power to be correlated. Thus, the relationship between voice power and negative content might additionally be an important aspect for research, particularly because at present there are conflicting ideas on how talking treatments for voice hearers, ranging from CBTp, compassion focused therapy, trauma focused therapy or more recently AVATAR-therapy could most effectively intervene in the voice hearers’ experience [30, 42, 46]. Interestingly in this study harming, self-harming behaviour and command hallucinations showed no correlations with voice power. These findings differed from the results of the COMMAND Trial [28], where voice power was found to be one of the best predictors of harmful compliance and a mediator of change. Nevertheless, it is important to note that the sample in this study differed from that of the sample in the COMMAND TRIAL in which participants had F2 or F3 diagnoses along with the experience of command voices. Furthermore, no relationship between voice power and overall severity (CGI-SCH) was found.

The main limitation of this study was that it focused on a subsection of the voice hearing population, namely those with an ICD 10 F2 diagnosis who were seeking treatment at the time of the study. Therefore, the findings of the study cannot be generalised to other voice hearing populations, for example healthy voice hearers or people with mood disorders or Post Traumatic Stress Disorders, etc. In addition, it offers no insight into the experiences of healthy voice hearers and indeed we did not investigate how many of our study participants identify as healthy voice hearers when they are not in crisis.

## Conclusions

A person’s perception of the power of the voice they hear is important and can be measured easily, validly, reliably, and efficiently with the Voice Power Differential Scale.

## Supporting information

S1 Dataset (SAV)
